# *Aspegillus terreus*: From Soil to Industry and Back

**DOI:** 10.3390/microorganisms8111655

**Published:** 2020-10-25

**Authors:** Maria Vassileva, Eligio Malusá, Bettina Eichler-Löbermann, Nikolay Vassilev

**Affiliations:** 1 Department of Chemical Engineering, University of Granada, C/Fuentenueva s/n, 18071 Granada, Spain; mvass82@yahoo.com; 2 Research Institute of Horticulture, 96-101 Skierniewice, Poland; eligio.malusa@inhort.pl; 3 CREA—Research Centre for Viticulture and Enology, via XXVIII Aprile 26, 31015 Conegliano, Italy; 4 Institute of Land Use, Faculty of Agriculture and Environmental Sciences, University of Rostock, 18051 Rostock, Germany; bettina.eichler@uni-rostock.de; 5 Institute of Biotechnology, University of Granada, 18071 Granada, Spain

**Keywords:** *Aspergillus terreus*, plant growth promotion, biocontrol, itaconic acid production, pathogenicity

## Abstract

*Aspergillus terreus* is an important saprophytic filamentous fungus that can be found in soils. Like many other soil microorganisms, *A. terreus* demonstrates multiple functions and offers various important metabolites, which can be used in different fields of human activity. The first application of *A. terreus* on an industrial level is the production of itaconic acid, which is now considered as one of the most important bioproducts in the Green Chemistry field. The general schemes for itaconic acid production have been studied, but in this mini-review some lines of future research are presented based on analysis of the published results. *A. terreus* is also intensively studied for its biocontrol activity and plant growth-promoting effect. However, this microorganism is also known to infect important crops such as, amongst others, rice, wheat, potato, sugar cane, maize, and soybean. It was suggested, however, that the balance between positive vs. negative effects is dependent on the soil-plant-inoculant dose system. *A. terreus* has frequently been described as an important human pathogen. Therefore, its safety manipulation in biotechnological processes for the production of itaconic acid and some drugs and its use in soil-plant systems should be carefully assessed. Some suggestions in this direction are discussed, particularly concerning the uses in crop production.

## 1. Introduction

According to the hologenome theory of evolution, all plants and animals are associated with abundant and diverse microbiota and this naturally selected cooperation is transmitted between generations thus playing important role in adaptation and survival of higher organisms [1]. During the last years, host-microbial cooperation has become one of the most dynamic issues in biotechnology research areas, particularly in plant science, trying to use the results from biomedical studies on human microbiome to better understand the function and role of the plant microbiome, since both are characterized by almost the same number of microorganisms [2]. Soil, particularly the rhizosphere, is the most potent source of microorganisms as it contains up to 10^11^ microbial cells per gram [3]. In soil-plant systems, microorganisms exert various types of functions ranging from neutral and beneficial to pathogenic [4]. Although fungi (10^5^–10^6^/g soil) and bacteria (10^7^–10^9^ /g soil) can be observed together in soil, bacteria are the most studied microorganisms [5]. Fungi, however, are reported to have an important role in bacterial activity creating hospitable microhabitat particularly in unfavourable environmental conditions, but also negatively affecting bacteria by secreting inhibitory metabolites [6]. Fungi play an important role in the functioning of soil-plant systems and particularly in essential processes such as stabilization of soil structure, organic matter decomposition, biogeochemical transformations, and mobility and bioavailability of nutrients [7]. Mycorrhizal fungi and filamentous fungi, such as *Aspergillus, Penicillium, Mucor*, and *Trichoderma* are amongst the most dominant and studied beneficial soil microorganisms forming with plant roots a special microhabitat called mycorhizosphere [8]. The typical examples of active soil filamentous fungi are the fungi belonging to the genus *Aspergillus* with *A. niger* and *A. flavus* being the most important species [9]. *Aspergillus terreus* appears to be less famous and studied, although it is a saprophytic fungus that could be found to reside in many soils. Apart of its soil functions, isolates of *A. terreus* are known for many applications in biotech industry as producers of itaconic acid, but also of a number of secondary metabolites such as the drugs lovastatin, terrein, asperfuranone, and cyclosporine A used as immunosuppressant, anti-cholesterol, anticancer, and other bioactive compounds [10,11,12]. However, in some cases *A. terreus* is known to produce tremorgenic mycotoxins, which are secondary metabolites that elicit either intermittent or sustained tremors in mammals [13], as well as to act as plant pathogen [14,15]. In this mini-review, we describe the multifaceted role of *A. terreus* as plant growth promoting and biocontrol microorganism and as itaconic acid producer because of the significant renewed interest in this microorganism within the Green Chemical Industry. Special attention is paid to possible solutions aimed at avoiding its detrimental effects in soil-plant systems.

## 2. *Aspergillus terreus* as Itaconic Acid Producer

Itaconic acid is one of the most promising metabolites produced from microorganisms and one of the top 12 building-block chemicals used in the chemical industry as declared by the US Department of Energy in the beginning of the 21st century [16]. The production of this dicarboxylic acid by *Aspergillus terreus* was described in the first half of the last century [17], but this capacity was also found in other fungal microorganisms such as *A. niger, Ustilago zeae*, and *Candida sp*. [18]. Metabolomics studies determined that itaconic acid production in *A. terreus* is achieved through the decarboxylation of the TCA cycle intermediate, *cis*-aconitate, by the enzyme *cis*-aconitate decarboxylase (CAD) [19,20]. 

As some other biotechnological production processes, the first large-scale itaconic acid manufacturing was based on surface fermentation of *A. terreus* in aluminium trays. Further studies found improved itaconic acid yield under submerged fermentation conditions, which is now the preferred mode of commercial scale production [21]. Due to its characteristics as an environmentally friendly alternative replacing petrochemical products traditionally used in polymer production demand is constantly increasing, with a corresponding worldwide volume of about 80,000 tons of itaconic acid [11,22]. During the last years, a renewed interest in itaconic acid production has been observed as an application of the principles of circular economy due to the use of growing substrates based on wastes, with an increase efficiency of the production process and consequent decrease of the production costs [23]. Here, we present the key achievements in this field mainly oriented towards yield improvement and price reduction of the final product grouping them according to the search of improved strains, new substrates, new fermentation and downstream processes. 

As for other microbial productions at the industrial level [24,25], one of the easiest and most attractive approaches to enhance the itaconic acid yield is optimization of the medium composition based on cheap substrates. The current price of 2 US dollars per kg of itaconic acid is calculated from fermentation production process based on glucose [26]. The high cost of glucose-based medium and the fact that production medium accounts for 60% of the operating costs in industrial fermentations, makes natural, widely available starchy or lignocellulosic substrates highly preferred [23,27]. Starch is one of the most applied polysaccharides as a substrate mainly because of its low cost, safety, and composition stability. Starch and starch hydrolysates have been successfully used in the production of organic acids including citric acid (the itaconic acid precursor) [28]. The composition of raw [29,30] and hydrolyzed starch [31] applied in itaconic acid production should be carefully assessed before its use bearing in mind that the presence of phosphorus and nitrogen may affect negatively or positively [32] fungal growth and acid production. Similarly, pre-treatment operations of lignocellulosic wastes by enzymes or acids can produce unwanted toxic compounds (mainly furfural, hydroxymethylfurfural, and some acids and metal ions), which can cause negative effects on the overall fermentation process [33]. Some recent studies demonstrated that the application of the biorefinery principles in the itaconic acid production on lignocellulosic materials is questionable [34]. However, agro-wastes can be used as a substrate in itaconic acid production in conditions of solid-state fermentation after optimization of some important parameters such as the need of substrate pretreatment, inoculum size and age, moisture level, need of additional medium components, aeration, and initial pH and pH control amongst others [35,36].

Studies with different sugars such as xylose, mannose, maltose, arabinose, and sucrose, alone or mixed, have shown their potential as substrates in *A. terreus* fermentation [37,38]. However, compared to 90–160 g itaconic acid/L, achieved on glucose medium [39,40], the acid production was lower than 36.4 g/L using the best substrate (maltose) and hence, economically unacceptable. Glycerol was recently studied as a potential substrate in itaconic acid production due to its accumulation as a side co-product in biodiesel production [41]. The final yield varied depending on the strain from 69.7 g/L itaconic acid achieved after 15 days of bio-process [42] to lower concentrations of 27.6 g/L [43] and 26.9 g/L in a 120-h production process [44]. 

Other medium components, such as manganese, calcium, iron, zinc, cobalt, and nickel affect the fungal morphology (pellets or branched mycelium) and the corresponding acid yield [26]. Nitrogen and phosphate concentration limiting or not the fungal growth and productivity is still a controversial point as well as the importance of pH, suggested as the triggering factor of itaconic acid biosynthesis [32].

Low yields of itaconic acid, including alternative sugars like xylose, can be improved applying various bio-process technological approaches. Production processes different from a simple single batch were used in studying the effect of medium components and cultivation parameters [45] but immobilized systems showed higher organic acid production rate in repeated-batch and continuous mode of production in comparison with free-living cells. For example, Kautola et al. [46,47,48] and Vassilev et al. [49] used successfully passively immobilized *A. terreus* on media with different sugars. Using a continuous mode bioprocess, a stable itaconic acid continuous production of 26 g/L was reported during 80 days in a 120-day packed-bed process on glucose [49] while, using sucrose, the production was 2-fold lower [45,48]. It should be noted that under packed-bed reactor conditions the aeration rate was difficult to control. Nemestóthy et al. [50] have shown the importance of better oxygen mass transfer: increasing oxygen supply rate and stirring *A. terreus* in bioreactors under submerged conditions increased the acid production from 1.27 g/L day to 4–26 g/L day. Following this line of studies, air-lift bioreactors have shown advantages in comparison with stirred bioreactors as the itaconic acid production increased from 0.48g/L h to 0.64 g/L h at lower power input [51]. An effective method for increasing itaconic acid production by **in**-situ reactive acid extraction was recently proposed [52]. In shake flasks the volumetric productivity changed from 0.72 to 0.91 g/L h after adding the extractant mixture of trioctylamine and the diluent isopropyl myristate. Genetic engineering using hosts different from *A. terreus* was proposed as a tool for enhancing the itaconic acid production [53]. Particularly *A. niger*, which is a potent producer of citric acid, was successfully tested as a model for metabolic reconstruction [54]. Under fed-batch fermentation conditions, the recombinant strain produced 71.4% more itaconic acid compared to the unmodified strain. Despite the low concentration of 7.2 g/L, this experiment showed the potential of recombinant strains in itaconic aid production. However, some experts in this field suggest that currently, the success of producing higher yields of itaconic acid heterologously by *A. niger* seems unrealistic [55]. 

## 3. *Aspergillus terreus* as a Part of the Soil-Plant Systems: Friend or Foe?

There are a number of reports on *A. terreus* clearly stimulating plant growth and health. Plant growth promoting metabolites such as indole-acetic acid, phenols and flavonoids were found in the production broth of *A. terreus* [56] and correlated with higher total chlorophyll content and increased shoot (79%) and root (27-fold) length in tomato plants treated with the fungus. Waqas et al. [57] reported that all studied sunflower growth parameters and yield were improved in the presence of *A. terreus*. In addition, sunflower plants, inoculated with *A. terreus*, and infected with *Sclerotium rolfsii*, showed resistance to stem-rot disease, which underlines the multifunctional characteristics of *A. terreus*. The low level of salicylic and jasmonic acids in *A. terreus*-sunflower system in comparison to control diseased plants demonstrated the positive effect of *A. terreus* to counteract biotic stress conditions. Similar multifunctional properties of soil isolates of *A. terreus* were confirmed in pot experiments with tomato [58]. *A. terreus* selected amongst 49 other microorganisms, was found to promote plant growth after seed treatment simultaneously reducing infection by *Pseudomonas syringe* pv. *tomato* through a systemic acquired resistant mechanism. According to gene expression analyses, this effect was associated to the Salicilic Acid-mediated pathway, because of overexpression of PR1 gene, while no changes were noted for several genes of the Jasmonic Acid-mediated signaling pathway. Growth of barley seedlings resulted to be promoted by addition of *A. terreus* strain isolated from saline soil because of its P-solubilization capacity [59]. Interaction of secondary metabolites (dihydroisocoumarins) produced by *A. terreus* with plant auxins could be also hypothesized as an additional mechanism of plant growth promotion [60]. 

*A. terreus* is better known as a biocontrol agent thus confirming the positive, plant growth promoting and plant health protecting role of many microorganisms belonging to the metabolically multifaceted genus *Aspergillus* [61]. Various *Aspergillus* spp. were proved efficient for their antifungal activity against *Fusarium sambucinum* and *Phytophthora erythroseptica* [62] in a dual culture method. Treatment timing and use of culture filtrates were suggested as important points in developing new strategies for *A. terreus* application. *A. terreus* produced antifungal compounds, found in the crude extract of the growing medium, which significantly reduced the charcoal rot pathogen *Macrophomina phaseolina* growth *in vitro* [63]. The antibiotic butyrolactone, isolated from culture filtrate of *A. terreus* was capable of supressing the phytopathogenic fungi *Botrytis cinerea, Rhizoctonia solani, Pythium ultimum, Phytophthora capsici,* and *Fusarium oxysporum* [64]. Similar, reduced damping-off disease effect on cucumber was observed after application of *A. terreus* in soil infested with *Pythium aphanidermatum* [65]. The reduced plant mortality (about 70 %), could be explained by the effect of *A. terreus* on *P. aphanidermatum* growth, oospore production, and hypha development as observed in experiments with culture filtrate of the antagonistic fungus. In this case, *A. terreus* did not have any effect on the growth parameters of cucumber used as the model plant. Similar results were reported by Halo et al. [66]. In both studies, enhanced activities of some enzymes such as cellulase [65] and glucanase [66] were measured, which were hypothesized to facilitate the destruction of the pathogen hyphae. Disintegration and 100% mortality of *Sclerotinia sclerotiorum* by *A. terreus* was documented *in vitro* by Melo et al. [67] when, after an abundant hyphal growth and sporulation of the mycoparasite on the sclerotial surface, the cell wall of the inner ring cells of the pathogen was destroyed. Finally, it should be noted that *A. terreus* was effective as biocontrol agent against *Biomphalaria alexandrina* snails [68].

Although *A. terreus* is a part of the soil microbiota and can be found in many types of soil, its role in promoting plant growth and improving plant health is controversial as it can affect crops either positively or negatively. The latter statement is based on studies concerning both the effects on plants and animals, including humans [69,70]. It should be mentioned that *A. terreus* is the direct cause of damaging of important crops such as rice, wheat, potato, sugar cane, maize, and soybean through seed and overall plant contamination [14,71]. Amongst the numerous genome-analysed secondary metabolites potentially produced by *A. terreus*, terrein was relatively the most studied [72,73]. This metabolite is responsible for inhibition of seed germination and plant growth, provokes plant surfaces damages, and also inhibits the growth of competitors thus facilitating the fungal invasion in the respective environmental niche [74]. However, this negative effect could be transformed into a beneficial one through the probiotic approach (see below). Such approach could also allow to overcome the worst effect of *A. terreus* which can occur after its introduction into the human body where, similarly to other members of the genus *Aspergillus*, it can cause aspergillosis infection with a high level of mortality particularly in immunocompromised patients [75]. However, it has been also proved that *A. terreus* could be useful for the biosynthesis of taxol, a potent anticancer drug, which production was enhanced by the interaction of the fungus with endophytes of an indigenous African plant, *Podocarpus gracilior* (bastard yellowwood) [76].

How can we cope with this dual behaviour of *A. terreus* and how can we use its plant beneficial properties without the risk of damaging plants and preventing the entrance of its spores into the food chain? One of the most attractive alternatives is the use of the microbial metabolites, which are beneficial for plant growth and health [77,78]. These metabolites can be purified or simply sterilized as shown with other plant beneficial microorganisms. Culture filtrates of *A. terreus*, free of mycelia and spores are reported to improve plant growth characteristics and plant health. For example, fermentation filtrate of *A. terreus* was found to significantly reduce spore production of *P. aphanidermatum* compared to the control and simultaneously increased electrolyte leaching from the pathogen mycelium [66]. The main metabolic activity related to the above biocontrol activity was due to the presence of glucanase and siderophores in the filtrate. Applied at 20% (*v*/*v*), sterilized filtrate of 28-day culture broth of *A. terreus* reduced the damage caused by *Pythium ultimum* and *Fusarium* spp. causing tuber dry rot in potato [79,80]. Butyrolactone I derivatives produced by *A. terreus* resulted also active towards the phytopathogenic bacteria *Erwinia carotovora* and, to a lesser extent, against *Pseudomonas syringae* and *Botrytis cinerea*, two important pathogens of horticultural crops, including grape [81]. However, an interesting effect of these metabolites was the selective and strong inhibitory effect on the seedling development of a dicot species (*L. sativa*), similar to a common synthetic herbicide (acetochlor). The seedling growth inhibition of terrain, a major metabolite of *A. terreus,* was proved on *Mimosa pigra* and *Echinochloa crus-galli* [82] and was found to be concentration-dependent on radish (*Raphanus sativus*) [74], a biological activity which could be useful for weeds control purposes. 

Another very attractive approach is to combine the P-solubilizing activity of *A. terreus* and the plant beneficial metabolite production. Itaconic acid is known as a moderate solubilizer of inorganic insoluble phosphates, functioning better than gluconic acid [83]. Recently, *A. terreus* was shown to solubilize animal bone char with high content of insoluble phosphate. Under optimized conditions of solid-state fermentation, the amount of soluble P in the medium reached 50% of the total P supplied as insoluble phosphate [35]. Applying the immobilized cell fermentation strategy, the same strain entrapped in polyurethane foam, solubilized almost 60% of the total P presented in the animal bone char in 48-h cycles in conditions of repeated-batch process [44]. Interestingly, *A. terreus* strains were able to solubilize zinc-P also under very saline conditions (up to 10% NaCl) [59]. Such kind of filtrates were recently proved as a safe tool for stimulating plant growth avoiding additional formulation of biofertilizers and further risk of their establishment and development in soil [84,85]. Finally, the capacity of *A. terreus* to produce potent cellulases [86] makes this fungus attractive for applications in the production of organic fertilizers using plant-based substrates or carbon-rich wastes or by-products (e.g. brown coal) that are difficult to be utilized by the crops [87] as well as for the other white biotechnology processes. However, for all these possible applications, the optimization of fungal growth conditions should be performed to foster the specific metabolic profile that would be most appropriate for biocontrol or plant growth promotion uses [88,89]. 

Using the “metabolite” approach, products with different characteristics can be prepared depending on the soil properties and plant needs, combining filtrates from different compatible and non-compatible microorganisms. All metabolites in the filtrate should be determined and, if necessary, the metabolic profile could be managed by metabolic engineering tools to avoid the presence of potential toxic and undesired agents. Some downstream or in-situ techniques could be applied to selectively isolate the industrially important itaconic acid (or/and other metabolites) and use the rest of the fermentation broth as a plant growth promoter or biocontrol agent. Knowing well the beneficial metabolic characteristics of *A. terreus* would help in developing new technologies based on this microorganism. 

## 4. Conclusions and Future Trends

*A. terreus* can be accepted as a typical example of the claim that “Everything is everywhere” [90]. Due to its high significance in the medical, agricultural, and industrial fields of research and application, *A. terreus* has been extensively studied and new metabolites are constantly discovered. In this review, we have focused on the applications of *A. terreus* that are important for both the industry (mainly itaconic acid production) and crop production. Analysing the recent literature, it appears that well-known biotechnological tools (e.g. immobilized microbial cell systems or continuous mode bioprocesses) could solve the problem of itaconic acid production price – the main reason for the limited production worldwide. Metabolic and genetic engineering could improve itaconic acid production using the microorganism of choice (*A. terreus*) [91] and other industrially proven microorganisms [54]. On the other hand, some classical tools such as media optimization and wider application of different fermentation and immobilization techniques should be studied more rationally to yield an improved production process as shown in the production of other organic acids [92]. In the field of crop science and technology, the use of *A. terreus* is still controversial. It is clear that this soil filamentous fungus, frequently found also as endophytic, is a natural component of many soil-plants systems. It can promote plant growth and demonstrated strong biocontrol activity, but at the same time it could provoke great damages on food grains. The question is how to ensure the safe use of *A. terreus* plant beneficial properties and avoid its negative influence on crops. Based on our knowledge, various strategies can be developed, the most attractive of which is the use of the fungal metabolites as *A. terreus* role as a cross-kingdom pathogen should be also considered. However, further studies should also be performed on *A. terreus* in different soils to analyse the interactive behaviour of all components of the plant-microbiome community. 
